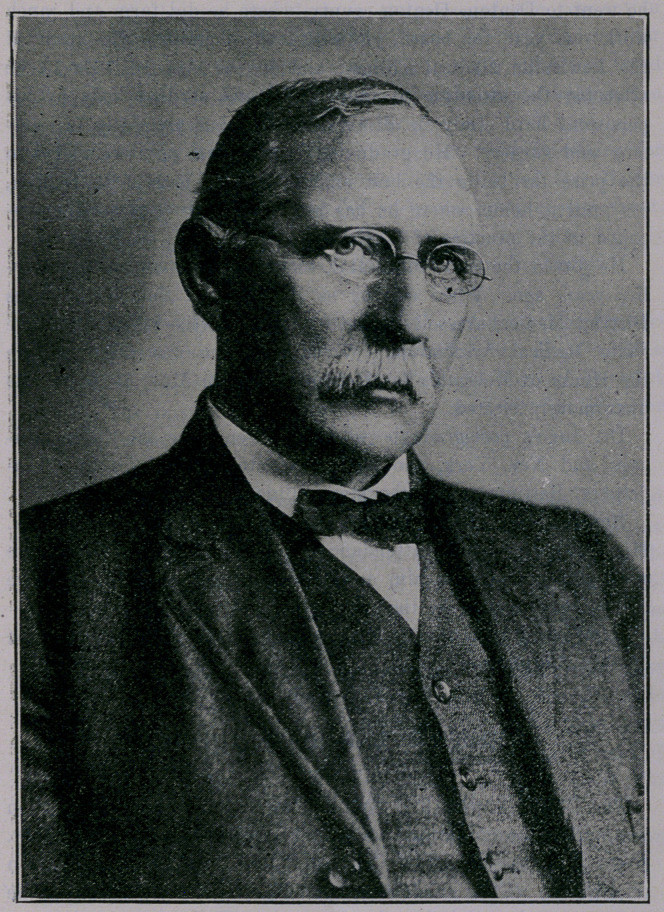# Pellagra

**Published:** 1916-06

**Authors:** 


### Pellagra.

Is the United States to become worse inflicted with pellagra than Italy? Are thousands upon thousands to be allowed to die every year because the country does not understand the disease, while the national and State governments sit supinely by and watch the death roll grow larger? Will the country submit to a curse that is already counting its victims by the hundreds in many States and that threatens to deplete the population even worse than war, and not make any organized effort to stop it?

The little that has been done to check pellagra has been at the individual expense of noble hearted, public-spirited physicians who have been studying the subject, but without government aid these men are unable to carry on the fight. In fact, it should not be expected that a few physicians should bear the brunt of a national scourge, which is already claiming victims in greater numbers than yellow fever, cholera, and the other "plagues" that formerly enlisted the aid of the government.

The small amount of $60,000 was spent last year in combating pellagra, while a few years ago more than $10,000,000 was used in stamping out the foot and mouth disease among the cattle of this country. Dr. K. H. Beall, of Fort Worth, who has made a close study of the subject says that at least 50,000 people have been killed in this country by pellagra, while 500,000 more have been made invalids by it. There were 661 deaths reported in Texas last year (and it is doubtful if half were reported), and the money loss to the State was not less than $1,500,000, due to pellagra, deaths and illness. The disease is increasing at an enormous rate, but not one thing has the State done to stop its ravages. Had the loss among cattle or hogs or grain or fruit been only half so heavy the Legislature would doubtless have been called in special session to take steps to stop it. We talk pathetically about war losses, but do practically nothing to check death losses from disease.

The national and State governments should come to a realization of the fact that it is as essential to protect citizens from the inroads of disease as from war. Why spend billions for defense against an imaginary foreign enemy and leave the people to die helplessly from the invasion of death-dealing diseases? The fact that pellagra victims die quietly, a few each day in each State, and that other suffer uncomplainingly, should not cause the nation and State to close their eyes to the importance of stopping it. A few citizens were sensationally killed by Mexicans at Columbus, New Mexico, and millions of dollars are now being spent to prevent another raid of that kind. A thousand will die in Texas this year from pellagra, and many times that number will suffer from the disease wounds. The life of a pellagra victim is worth just as much as the life of a resident of Columbus, and should be just as carefully safeguarded.

The time will come when the country that will negligently permit its citizens to die from disease will be regarded as no better than the country that will allow its people to be shot down by invading foes. An educational campaign will be required to bring this about, and it devolves on the physicians and the medical journals to take the lead. Legislators and congressmen must be shown that they are in a large measure the custodians of the lives of the people, and that they must be guarded as valuable assets of the country.

**Dr. J. M. Inge, President of State Medical Association.**

Dr. James M. Inge was born in Graves county, Kentucky, in February, 1852.

In 1858 his father moved to Texas for the purpose of making a new home with superior advantages in which to rear his growing family. They settled on a farm in Fannin county, Texas, where the subject of this writing lived with the family, worked on the farm, and attended such schools as the rural sections then afforded. In 1868, when he was sixteen years of age, he went to Denton, Denton county, where he did farm and ranch work one year for board, clothing and schooling. He attended the Lewisville Medical College, and in the class of 1872-73 he attracted the attention of the faculty and student body by his persistent hard study in all the branches, but especially in anatomy and surgery. He graduated in the class of 1874 and won the prize for being the best anatomist. He located in Denton, his present home, where he has been constantly and actively engaged in the practice of medicine and surgery up to this time.

He joined the State Medical Association of Texas about thirty-five years ago. When serving as President of the North Texas District Medical Association he was elected Vice-President of the State Medical Association. When his name was placed before the House of Delegates for President-elect in May, 1915, he was unanimously elected.

Dr. Inge's post-graduate work has been done mostly in Chicago and New York, though he has attended clinics in Philadelphia, Baltimore, and the Mayo clinic in Rochester, Minn.

But the doctor needs no introduction to the Texas physicians. His years of faithful attendance upon and labor for the State Association has made him a well known and much loved figure in the counsel halls of the Association.

A full write-up of Dr. Inge was given in June, 1915, issue of this JOURNAL.

## Figures and Tables

**Figure f1:**